# Expression of regulatory and executor proteins of apoptosis in odontogenic keratocyst: a systematic review

**DOI:** 10.4317/medoral.26973

**Published:** 2025-08-16

**Authors:** René Martínez-Flores, Matías Muñoz-Orrego, Constanza Marín-Márquez, Lauren Frenzel Schuch, Felipe Martins Silveira, Ronell Bologna-Molina, Sven Eric Niklander

**Affiliations:** 1 Unit of Oral Pathology and Medicine, Dentistry Faculty, Universidad Andres Bello, Viña del Mar, Chile; 2 Dentistry Faculty, Universidad Andres Bello, Viña del Mar, Chile; 3 Faculty of Dentistry and Rehabilitation Sciences, Universidad San Sebastián, Puerto Montt, Chile; 4 Department of Diagnosis in Pathology and Oral Medicine, Dentistry Faculty, Universidad de la República, Montevideo, Uruguay

## Abstract

**Background:**

The odontogenic keratocyst (OKC) corresponds to the third most common odontogenic cyst of the maxillary bones, originating from the dental lamina or its remnants. Apoptosis dysregulation, due to an imbalance between anti-apoptotic and proapoptotic proteins, has been proposed as a promoter for the development and progression of OKC. This study aimed to conduct a systematic review to synthesize the current knowledge on effector proteins of the intrinsic and extrinsic pathways, and executor proteins of apoptosis in OKC and compare their expression to other odontogenic cysts and tumors.

**Material and Methods:**

Primary studies were searched in PubMed, Scopus, and Web of Science databases, following the recommendations of PRISMA. Inclusion criteria were articles in English reporting the expression of at least two apoptosis-related proteins in OKC, studies using human tissues, descriptive retrospective case series, or *in vitro* assays.

**Results:**

Seven articles met the inclusion criteria and were considered for data extraction and analysis. Of the selected articles, six studied proteins related to the regulation of the intrinsic pathway of apoptosis, all reporting the immunohistochemical expression of Bcl-2 and BAX. Only one study reported the immunohistochemical expression of proteins related to the regulation of the extrinsic pathway, specifically Fas and FasL. Regarding apoptosis execution proteins, only one article characterized the immunohistochemical expression of caspases, specifically caspase-3.

**Conclusions:**

OKC expresses proteins related to apoptosis regulation similar to other aggressive odontogenic lesions, such as ameloblastoma. This suggests that apoptosis dysregulation may be essential in its development and progression.

** Key words:**Odontogenic keratocyst, odontogenic tumor, apoptosis, odontogenic cysts.

## Introduction

Odontogenic cysts correspond to a group of lesions originating from tissues involved in dental formation once odontogenesis is completed. They are classified into inflammatory and developmental cysts (1,2). The odontogenic keratocyst (OKC) belongs to the latter group. Of all odontogenic cysts, OKC is generally stated as the third most common cyst of the jaws, originating from the dental lamina or its remnants. It predominantly affects the mandible of men between the second and third decades of life. Usually, it manifests as a sporadic solitary lesion, but multiple lesions do also occur as part of Gorlin-Goltz Syndrome (GGS) or Nevoid Basal Cell Carcinoma Syndrome (NBCCS). Despite being a benign cystic lesion, it can destroy large portions of bone due to its locally infiltrative growth, which partly explains its high recurrence rate (3). Similar to basal cell carcinoma, both sporadic and multiple OKC in GGS are associated with activation of the sonic hedgehog signaling pathway, secondary to a somatic mutation in the human homologue 1 of the Drosophila Patched gene (PTCH1). This event has been proposed as the primary mechanism underlying OKC development, by promoting cell cycle progression and preventing apoptosis (4).

**Figure 1:**
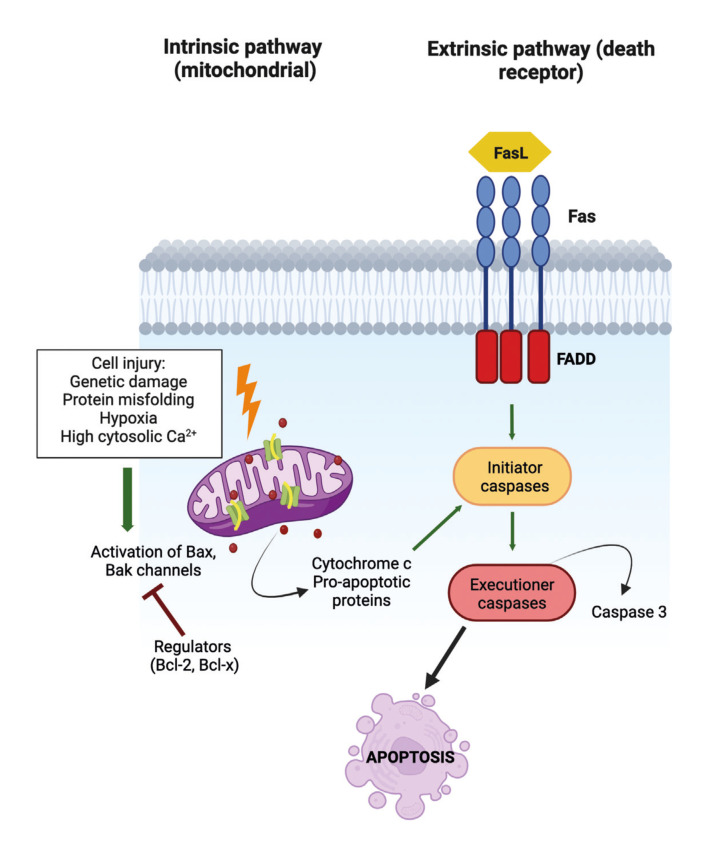
Apoptosis signaling pathways. The intrinsic pathway is triggered by internal stimuli that cause cell injury, increasing the permeability of mitochondria and releasing cytochrome C into the cytosol. Cytochrome C activates initiator caspases that activate downstream caspases, such as caspase 3, causing cell death. Two groups of proteins regulate the intrinsic pathway called anti-apoptotic (e.g., Bcl-2, Bcl-XL and Mcl-1) and proapoptotic proteins (e.g., Bak, Bax, Bad, Bcl-Xs, Bid, Bik, Bim and Hrk). The balance between the actions of anti and proapoptotic proteins determines how apoptosis is introduced. The extrinsic pathway begins when a death ligand (FasL) binds to a death receptor, such as tumour necrosis factor receptor (TNFR1) or Fas (CD95). These receptors have a death domain that requires adaptor proteins, such as Fas-associated death domain (FADD), activating initiator caspases that can cleave other downstream or executioner caspases and initiate apoptosis. Created with BioRender.com.

In vertebrates, apoptosis is important for proper development, maintenance of tissue homeostasis, and cancer prevention. Apoptotic cell death is initiated by internal or external stimuli, and is mediated through two distinct pathways: the intrinsic (mitochondria-mediated) and the extrinsic (death receptor-mediated). These pathways ultimately converge in the activation of cysteine-aspartic proteases called caspases, executor enzymes responsible for degrading cellular components and facilitating their subsequent removal by phagocytes (5) (Fig. 1).

The imbalance between anti-apoptotic and proapoptotic proteins can induce apoptosis dysregulation, promoting oncogenesis and tumorigenesis (6). The expression of apoptosis-related proteins has been studied in various human tumors; however, their role in the development and progression of odontogenic lesions such as OKC is limited (7).

The treatment of OKC is challenging and is still a debated topic, as recurrence of OKCs ranges from 2.5 to 62.5% depending on the treatment of choice (8). To aid in reducing its recurrence rate, different adjunctive therapies have been used, including the application of Carnoy’s solution (CS) . Despite the use of CS in combination with enucleation and curretage has reduced the recurrence rate of OKC significantly (9) (although with a significant risk of causing paresthesia to the inferior alveolar nerve), CS has been banned by the Food and Drug Administration (FDA), as CS has chloroform, which is a known carcinogenic. This has led to the search of new topical adjunctive agents to reduce recurrence. 5-Fluorouracil (5FU), an antimetabolite agent that targets DNA synthesis causing apoptosis of rapidly diving cells, has been increasingly used on the surgical bed of OKC, showing promising results in terms of reducing recurrence and paresthesia (9,10). Despite being the induction of apoptosis the main way of action of 5FU, little is known about the regulation of apoptosis in OKC. Thus, this study aimed to conduct a systematic review (SR) of the literature on the regulatory proteins of the intrinsic and extrinsic pathways, as well as executor proteins of apoptosis in OKC, to consolidate current knowledge and identify aspects of apoptosis in OKC that remain unexplored. This is of importance considering that the apoptotic pathway is the therapeutic target of 5-FU therapy, and little is known about its expression in OKC.

## Material and Methods

- Research question

Which key regulatory proteins involved in the intrinsic, extrinsic, and executioner apoptotic pathways are differentially expressed in OKC, and how do these patterns compare to other odontogenic cysts and tumors?

- Study design, protocol and registration

This systematic review was conducted following the recommendations from PRISMA 2020 (Preferred Reporting Items for Systematic Reviews and Meta-Analyses) (11). Our research protocol was registered on the Open Science Framework (OSF; osf.io/hy7zb). Our SR followed the Population, Exposition, Comparators, Outcomes and Study Design (PECOS) framework to determine the inclusion criteria, identifying key points as follows: population- human tissue, exposition- odontogenic keratocysts, comparators- any other odontogenic lesion, outcomes- expression of a minimum of two apoptosis-related proteins, and study design- primary investigations such as case series or *in vitro* assays).

- Eligibility criteria

Articles in English reporting the expression of at least two apoptosis-related proteins in OKC, studies using human tissues, descriptive retrospective case series studies or *in vitro* assays were included. Studies primarily reporting cell cycle-related proteins or proliferation markers, whole-genome association studies, studies including only epithelial odontogenic tumors, and isolated case reports were excluded.

- Information sources and search strategy

To identify potentially relevant articles, the following databases were selected: PubMed, Scopus, and Web of Science. The search was independently conducted by RMF and MMO between the 15th and 30th of March, 2023. The search terms used were "apoptosis," "cell cycle," "odontogenic cyst," and "odontogenic lesions," which were combined using the boolean operators "AND" and "OR" following the structure: ((apoptosis) OR (cell cycle)) AND ((odontogenic cyst) OR (odontogenic lesion)). In addition, a manual search of the relevant references of the included primary studies was conducted.

- Selection of Sources of Evidence

Article selection was performed independently by RMF and MMO and recorded in an Excel spreadsheet developed for this review. Duplicate articles were removed, while the remaining articles were screened by title and abstracts. To reduce bias in article selection, full-text review was performed by two reviewers (MMO and RMF). In cases of disagreement, a third (SEN) and fourth reviewer (CMM) acted as judges and made the final desition.

- Data collection and charting

Data extraction was independently conducted by RMF, MMO, SEN and CMM. In order to reduce bias in the process of data extraction, data of each article was extracted by at least two reviewers. The following information obtained from the selected primary articles was recorded in an excel spreadsheet developed for this review: first author's last name, year of publication, country, study group, sample size, control group, molecular biology technique used, type of apoptosis-related protein and its role, expression pattern and distribution in tissue, method of protein expression evaluation, and significant findings of the studies. To assess study heterogeinity, the different methodologies employed by the different studies were also registered.

- Quality assessment

The quality of the included case series studies was evaluated using the Joanna Briggs Institute (JBI) Critical Appraisal Checklist for Case Series (12). This checklist consists of ten items that assess key aspects of study design and reporting. Each item was rated as 'Yes,' 'No,' 'Unclear,' or 'Not Applicable,' and the overall quality of each study was determined by the proportion of items rated as 'Yes.'

## Results

- Selection of Evidence Sources

**Figure 2:**
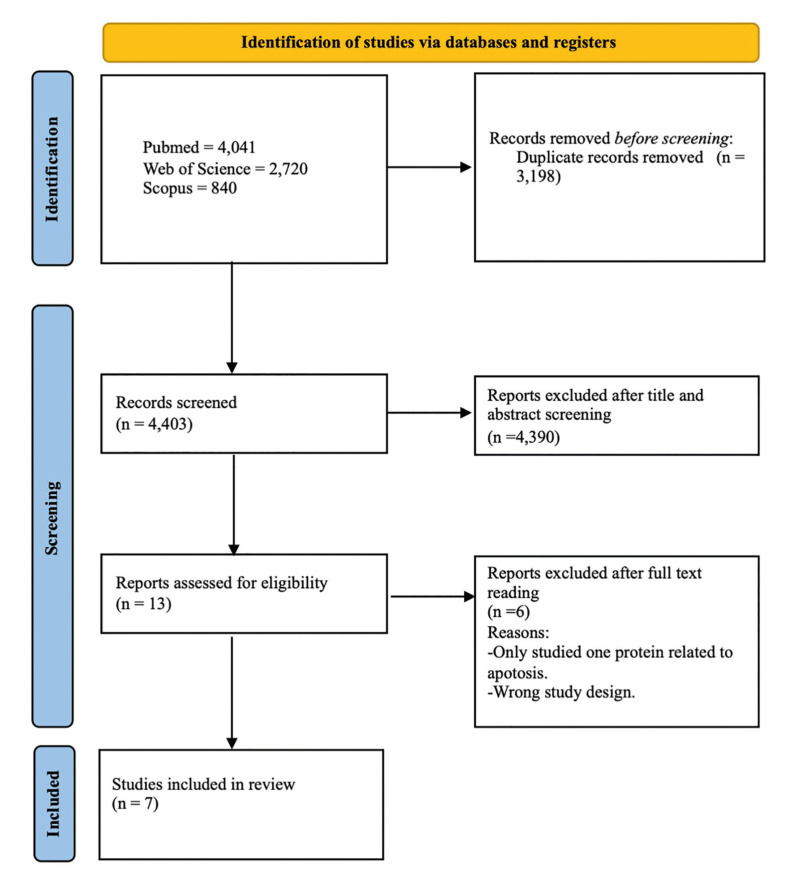
PRISMA flowchart of the article selection process.

The search strategy yielded a total of 7,601 records: 4,041 articles from PubMed, 2,720 from Web of Science, and 840 from Scopus. Of these, 3,198 were duplicates and were excluded, leaving 4,403 articles for the review stage. After reviewing titles and abstracts, only 13 records were initially selected, and 7 of these met the eligibility criteria and were included for data extraction (Fig. 2).

The seven articles included in this review consisted of retrospective descriptive case series. All of them reported using immunohistochemistry from formaly-fixed paraffin embedded (FFPE) tissue samples. The methodology for evaluating the immunohistochemical expression of proteins varied among studies. Three studies used different semi-quantitative evaluation schemes (3,6,13), while four utilized quantitative systems for the estimation of proportions (7,14,15). Additionally, one study reported the ratio between immunopositive cells for Bcl-2 and Bax, and another reported the correlation between Bcl-2 and Bax expression (6) (Table 1).

- Critical Appraisal within Sources of Evidence

Across the studies, most criteria were consistently met. All included studies demonstrated clear inclusion criteria, reliable measurement of the condition, and appropriate statistical analysis. However, variations were observed in the consecutive and complete inclusion of participants. All details and specific ratings for each study are presented in Supplement 1.

- Results of Sources of Evidence and Data Synthesis

All seven included articles considered sporadic odontogenic keratocysts (OKCs/S) as the study group. Regarding the control group, three included ameloblastomas (AM) (6,7,14), one included adenomatoid odontogenic tumors (AOT) (6), two included radicular cysts (RCs) (14,16), one included dentigerous cysts (DCs) (16), one included orthokeratinized odontogenic cysts (OOCs) (15), three included syndromic odontogenic keratocysts (OKCs/Si) (7,13,16), one corresponded to a paired study comparing sporadic OKCs before and after decompressive treatment (3) , and one considered recurrent sporadic OKCs (OKCs/RS) (13) (Table 1).

Of the seven selected articles, six examined proteins associated with the regulation of the intrinsic apoptosis pathway, with all reported the immunoexpression of Bcl-2 and Bax (3,6,7,14-16). Only one study reported the immunohistochemical expression of proteins related to the regulation of the extrinsic pathway, specifically the proteins Fas and FasL (13). Regarding proteins related to the execution of apoptosis, only one article characterized the immunohistochemical expression of caspase-3 (13) (Table 1).

Bcl-2 was expressed in a cytoplasmic pattern, with predominant expression in the basal stratum (3,6,7,14-16). Bax was expressed in a cytoplasmic and nuclear pattern, and the distribution of immunopositive cells was observed from the basal to the superficial stratum, with variations between studies. Regarding proteins associated with the extrinsic pathway, Fas and FasL presented a cytoplasmic expression pattern, with FAS positive cells located throughout the whole epithelial thickness and FasL positive cells in the basal/parabasal layers (13). Caspase-3 was expressed both in cytoplasm and the nucleus, with immunopositive cells distributed from the basal to the superficial stratum (13).

As for the expression of Bcl-2, one study reported higher expression in OKCs/Si compared to OKCs/S (16). In contrast, another study did not observe differences between both groups (7). In one study, Bcl-2 expression was compared before and after decompression. Null, moderate, and intense expression were observed in 33.3%, 52.4%, and 14.3% of cases before and after decompression, respectively. These differences were not statistically significant (3). (Table 2).

The literature also compared the expression of Bcl-2 in OKCs/S and OKCs/Si with three other odontogenic cysts. Two articles reported a higher expression of Bcl-2 in OKCs/S compared to RCs (14,15), and a higher expression in OKCs/Si compared to RCs (16). Another study reported higher expression of Bcl-2 in OKCs/S and syndromic OKCs/Si compared to DCs (16). Finally, one study observed Bcl-2 expression in all cases of OKCs/S and no expression in all analyzed cases of OOC (15).

The expression of Bcl-2 in the cystic epithelium was also compared with the neoplastic epithelium of odontogenic tumors. Lower expression of Bcl-2 was described in OKCs/S compared to conventional ameloblastoma (AMc) and unicystic ameloblastoma (UA) (7). In contrast, a study that used a semi-quantitative evaluation system of Bcl-2, found no statistically significant differences between OKCs/S, AMc and AOT (6) (Table 2).

Regarding Bax expression, one study did not observe statistically significant differences when comparing the expression in OKCs/S and OKCs/Si (7). Another study assessed the impact of decompression on Bax expression. Negative, mild, moderate, and intense expression was reported in 23.8%, 23.8%, 42.9%, and 9.5% of the cases respectively before decompressive treatment, with no significant changes after decompression (3) (Table 2).

Bax expression was also compared between OKCs/S and OKCs/Si with three other odontogenic cysts. One article reported significantly higher Bax expression in OKCs/S compared to RCs (16), while another found lower expression in OKCs/S when compared with RCs (14). Bax expression was also significantly higher in OKCs/S and OKCs/Si compared to DCs (16). No statistically significant differences were found when comparing mean Bax expression in OKCs/S to OOCs (Table 2) (15).

Bax expression in cystic epithelium was also compared with the neoplastic epithelium of odontogenic tumors. One study reported no statistically significant differences when comparing Bax expression between OKCs/S and AMc (14). A similar result was reported in another study that compared Bax expression in OKCs/S and OKCs/Si with both AMc and UA (7). In contrast, a study employing a semi-quantitative evaluation system for Bax expression in OKCs/S, AMc, and AOTs found no statistically significant differences between the groups (6) (Table 2).

One study evaluated the Bcl-2/Bax ratio in OKCs/S and OKCs defining the predominance of Bcl-2 or Bax when the result of the division of the average percentages of protein expression was >1 or <1, respectively. A predominance of Bax over Bcl-2 was reported in 80% and 66.7% of sporadic OKCs and syndromic OKCs respectively.

Only one study evaluated the expression of proteins related to the extrinsic and executioner pathways of apoptosis in OKCs using a semi-quantitative assessment method. All studied cases of OKCs/S, OKCs/Si, and OKCs/RS expressed Fas, with significantly higher expression in OKCs/Si than in OKCs/S. 41,7%, 77%, and 60% of OKCs/S, OKCs/Si, and OKCs/RS cases respectively, were positive for FasL, with not statistically significant differences between the groups. All studied cases of OKCs were positive for caspase-3, reporting marginal or intermediate positive expression in the different study groups, differences that were not statistically significant (13) (Table 2).

## Discussion

Apoptosis is the most studied form of cell death, and the primary mode of cell death involved in embryonic development and tissue homeostasis. The key to its regulation lies in the delicate balance between pro- and anti-apoptotic proteins, such as those involved in the intrinsic pathway of apoptosis, which was the most studied pathway in this review. While the former induces cells to escape apoptosis permanently (e.g., Bcl-2), the latter allows cells to initiate the irreversible path to death, as is the case with BAX, Bak, BAD, and BID (17-21).

The large number of interacting proteins involved in the regulation and execution of apoptosis makes it a complex biological phenomenon. An example is the interaction between Bcl-2 and BAX. Bcl-2 inhibits the function of BAX, preventing it from forming pores in the mitochondrial outer membrane, thus avoiding the efflux of proapoptotic factors such as Ca+2 and cytochrome C from the intermembrane space of the mitochondria to the cytosol. This, in turn, prevents the activation of initiator caspase 9, thereby preventing the activation of executioner caspases 7, 6, and 3, which are responsible for the cleavage of protein substrates such as cytoskeletal proteins and the DNA-nuclease inhibitor. In this way, biochemical and morphological changes, and typical phagocytic elimination are avoided (17-21).

This review revealed that Bcl-2 is expressed higher in AM than in OKCs (7,14) , but Bcl-2 is expressed significantly higher in OKCs compared to other odontogenic cysts, such as OOCs, DCs, and RCs (14-16). This suggests that the anti-apoptotic function of Bcl-2 may play an essential role in the development and progression of AM and OKC. These two lesions cause significant destruction in the maxillary bones and are well known for their high recurrence rate if improperly removed (6). On the other hand, the literature shows that both OKC/S and OKC/Si have similar BAX expression levels, but higher when compared to DCs and RCs (16). When compared to odontogenic tumors, OKC/S has similar BAX expression to AMc, UAs, and AOTs, suggesting that it plays an essential role in maintaining cell populations in odontogenic lesions with aggressive and non-aggressive local growth (6,7,14). However, attributing a lesion's biological and clinical behavior solely to the overexpression of just one apoptosis-regulating protein seems insufficient, given the complexity of the biological bases underlying its development (1,4).

By calculating the ratio of the mean expression of Bcl-2/BAX, a study reported a ratio of Bcl2/BAX <1 in most cases of OKC/S, OKC/Si, AMc, and UA, meaning a predominance of BAX over Bcl-2. This suggests that the expression of proteins of the Bcl-2 family would be shifted towards the induction of apoptosis. Nevertheless, this is not necessarily so. For example, BAX predominance could be related to the differentiation and maturation of the odontogenic epithelium, rather than having an essential role in the induction of apoptosis (6,7,14). Also, a predominance of proapoptotic proteins does not necessarily guarantee the activation of initiator and executioner caspases, as shown by a study where no significant differences in caspase-3 expression levels (one of the three executioner caspases responsible for the main catalytic events underlying apoptosis) were found between OKC/S, OKC/RS, and OKC/Si (13).

In chronic inflammation, the balance between pro-apoptotic and anti-apoptotic proteins is crucial for eliminating damaged or dysfunctional cells without triggering excessive inflammation (22). It has been demonstrated that RC predominantly express Bax over BCL-2, supporting the notion that within inflammatory periapical lesions, these Bcl-2 family members regulate apoptosis through a balance that contributes to cell proliferation and epithelial cell death (23). These findings aligns with Soluk *et al*. (14), who observed higher Bax expression in RC compared to OKC/S, but is opposed to the findings reported by Kolář *et al*. (24), who reported lower Bax expression in RC compared to a combined cohort of OKC/S and OKC/Si cases. This discrepancies might be explained by alterations in the Sonic Hedgehog sgnaling pathway. Alteration in this pathway can be caused by a somatic mutation in PTCH1, which is widely recognized and proposed as the main mechanism underlying the tumorigenesis of OKC. Notably, PTCH1 mutations lead to Gli1 overexpression, which promotes cell cycle progression and enhances the transcription of anti-apoptotic proteins such as Bcl-2, further contributing to apoptosis resistance. Although it appears to be more frequent in OKCs associated with the basal cell nevus syndrome, it is also reported in both multiple and solitary lesions, (1,2,4,25,26).

Our findings highlight that OKC pathogenesis not only involves increased expression of cell cycle-promoting proteins (secondary to PTCH1 mutations and Hedgehog pathway activation), but also reveals a significant role for apoptosis regulation in the epithelial lining of the cyst. This observation aligns with previous suggestions (27) that OKC growth is influenced by a dual mechanism: apoptosis occurring at a high rate in the superficial layer of the epithelial lining; and Bcl-2 overexpression in the basal layer, which inhibits apoptosis and facilitates cell proliferation. This balance appears to regulate the high proliferation rate driven by Hedgehog pathway activation.

**Figure 3:**
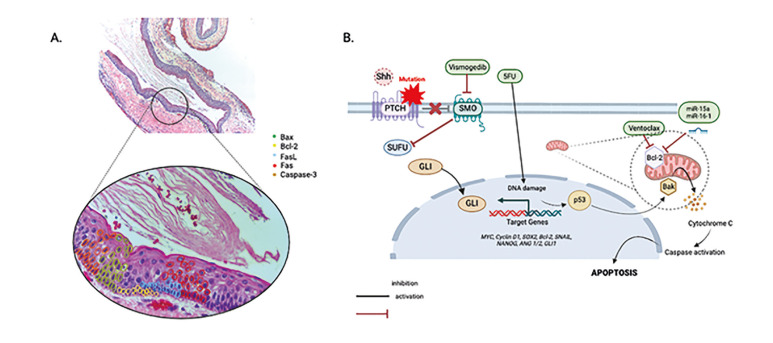
Molecular insights into apoptosis regulation and therapeutic targets in Odontogenic Keratocyst A) Photomicrograph of an OKC, highlithing the expression and distrinution of the markers found in the articles included in this study. B) Proposed molecular mechanism of apoptosis regulation and novel therapeutic targets in OKC. PTCH1 mutations result in constitutive activation of the Sonic hedgehog (SHH) pathway, allowing SMO to promote Gli1 overexpression. Gli1 enhances the transcription of cell cycle regulators and anti-apoptotic proteins such as Bcl-2, contributing to apoptosis resistance and promoting OKC progression. Bcl-2 overexpression inhibits Bak, preventing mitochondrial membrane permeabilization and blocking the release of cytochrome c, a key apoptotic signal that activates caspases and promotes cell death. Vismodegib inhibits SMO, reducing Gli1-mediated transcription and thereby limiting Bcl-2 overexpression. 5-Fluorouracil (5-FU) induces DNA damage, triggering p53 activation, which enhances the expression of pro-apoptotic proteins like Bax and promotes apoptosis. Venetoclax, a Bcl-2 inhibitor, directly counteracts Bcl-2 function, restoring mitochondrial permeability and promoting cytochrome c release. miR-15a/miR-16-1 mimetics downregulate Bcl-2 expression, further promoting apoptosis through mitochondrial signaling. Created with BioRender.com.

Our findings also suggest that the intrinsic apoptotic pathway would play a more significant role than the extrinsic pathway in OKC pathogenesis, evidenced by the frequent detection of Bcl-2 and Bax expression patterns (Fig. 3). Nevertheless, additional studies employing quantitative molecular techniques, such as TUNEL assays, are necessary to confirm these observations and further elucidate the molecular mechanisms involved.

Given its aggressive clinical course and high recurrence rate, a targeted therapy may offers decreased post-oprerative morbidity, less recurrence and thus, lower risk of reoperation. The use of 5FU as a topical adjunctive agent has shown promising results reducing recurrence and paresthesia (28). Our results suggest that, in addition to inhibiting cell proliferation, apoptosis could also be induced, thus a combination therapy targeting both the Sonic hedgehog (SHH) pathway and Bcl-2 overexpression could provide a novel therapeutic approach for large, recurrent or multiple OKCs. Specifically, the combination of Vismodegib, an SHH pathway inhibitor, with an Bcl-2 inhibitors, such as Venetoclax (both approved by the FDA) (29), or novel Bcl-2 inhibitors, such us miR-15a/miR-16-1 mimetics (30), could enhance the expected results (Fig. 3).

This dual-targeted strategy addresses the two key mechanisms driving the progression of OKCs and may improve treatment outcomes, particularly in lesions with PTCH1 mutations.

Another concern raised in this review is the methodological heterogeneity regarding the systems for evaluating protein immunoreactivity declared in the different studies. While quantitative evaluation systems allow for obtaining objective data (7,14,16), semi-quantitative evaluation schemes may lead to underestimation or overestimation of the expression of a particular protein (3,6,13).

This review is an example of the complex regulation of apoptosis in OKC and other odontogenic lesions and how little is known about this in odontogenic lesions. Only seven records complied with the inclusion criteria and were included in this review, and only one of them assessed the expresssion of Fas, FasL and Caspase-3, while six assessed the expression of BAX and Bcl-2. Although the significant diversity in the expression of these proteins may be related to the variability in the clinical and biological behavior of these lesions, methodological heterogeneity complicates the comparison of the results obtained in the reviewed studies, which is a limitation of this study. Therefore, there is a need to promote further research studying proteins involved in the different stages underlying the pathogenesis of apoptosis. Additionally, the use of quantitative methods for evaluating protein expression when using immunohistochemical techniques, as well as the incorporation of other molecular biology techniques that allow determining the actual presence of apoptotic cells with DNA fragmentation, such as the TUNEL assay, should be encouraged.

## Conclusions

OKC expresses proteins related to the intrinsic, extrinsic, and caspase pathways of apoptosis in a profile similar to other aggressive odontogenic lesions such as AM, suggesting that the dysregulation of apoptosis may play an essential role in its development and progression. However, further research is needed to better understand the molecular biology of the apoptosis pathway in OKC. New studies involving special techniques to assess apoptotic cells, such as the TUNEL assay, or directed to assess initiator and effector caspases through immunohistochemistry, would improve the existing knowledge regarding the role of BCL-2 family proteins in OKC development.

## Figures and Tables

**Table 1:** Summary of selected articles.

First autor, year	Country	Study group (n)	Control group (n)	Pathway	Proteins	Evaluation
Escobar et al. (2023) (7).	Chile	18 OKC/S (n=18)	OKC/Si (n=15)	Intrinsic	Bcl-2 y Bax	Quantitative
			AMc (n=18)			
			UA (n=15)			
Trujillo-González et al. (2022) (3).	Venezuela	OKC/S (n=21)*	OKC/S (n=21)**	Intrinsic	Bcl-2 y Bax	Semi- Quantitative
Tenorio et al. (2018) (6).	Brazil	OKC/S (n=20)	AM (n=20)	Intrinsic	Bcl-2 y Bax	Semi- Quantitative
			AOT (n=20)			
Tekkeşin et al. (2012) (14).	Turkey	OKC/S (n=20)	RC (n=20)	Intrinsic	Bcl-2 y Bax	Quantitative
			AM (n=20)			
Rangiani et al. (2009) (15).	Iran	OKC/S (n=28)	OOC (n=9)	Intrinsic	Bcl-2 y Bax	Quantitative
Kolář et al. (2006) (16).	Czech Republic	OKC/S (n=39)	OKC/Si (n=18)	Intrinsic	Bcl-2 y Bax	Quantitative
			RC (n=29)			
			DC (n=10)			
Kimi et al. (2001) (13)	Japan	OKC/S (n=24)	OKC/RS (n=10)	Extrinsic	Fas y FasL	Semi- Quantitative
			OKC/Si (n=9)	Executor	Caspase-3	

OKC/S: Sporadic odontogenic keratocyst; OKC/Si: Syndromic odontogenic keratocyst; OKC/RS: Recurrent sporadic odontogenic keratocyst; OOC: Orthokeratinized odontogenic cyst; RC: Radicular cyst; DC: Dentigerous cyst; AMc: ameloblastoma, conventional; UA: ameloblastoma, unicystic; AOT: Adenomatoid odontogenic tumor.

**Table 2:** Expression of Bcl-2, Bax, Fas, FasL, and Caspase-3 in odontogenic lesions.

Protein	Immunoxpression pattern	Expression and distribution	Summary of protein expression in study and control group
Bcl-2	Cytoplasmic (3, 6, 7, 14-16)	Basal (7, 16) Basal y parabasal (3, 6, 15) The entire epithelial thickness (14)	Higher expression in OKC/Si than OKC/S (16)
			Higher expression in OKC/S than RC (16)
			Higher expression in OKC/S and OKC/Si than DC (14)
			Higher expression in OKC/S than OOC (16)
			Lower expression in OKC/S and OKC/Si than AMc and UA (7)
			Lower expression in OKC/S than AMc (14)
			No significant differences between OKC/S and OKC/Si (7)
			No significant differences between OKC/S before and after decompression (3)
			No significant differences between OKC/S and AMc and AOT (6)
Bax	Cytoplasmic (3, 6, 7, 14-16) Nuclear (3, 6, 14, 16)	The entire epithelial thickness (3, 6, 14, 15) Basal y parabasal (7, 16)	Higher expression in OKC/Si than RC (16)
			Higher expression in OKC/S and OKC/Si than DC (16)
			Lower expression in OKC/S tan RC (14)
			No significant differences between OKC/S and OKC/Si (7)
			No significant differences between OKC/S before and after decompression (3)
			No significant differences between OKC/S and AMc (7, 14), UA (7) and AOT (6)
Fas	Membrane and cytoplasmic (13)	From parabasal to superficial (13)	Higher expression in OKC/Si than OKC/S (13)
FasL	Membrane and cytoplasmic (13)	Basal and parabasal (13)	No significant differences between OKC/S, OKC/RS and OKC/Si (13)
Caspase-3	Cytoplasmic and nuclear (13)	Basal, parabasal and superficial (13)	No significant differences between OKC/S, OKC/RS and OKC/Si (13)

OKC/S: Sporadic odontogenic keratocyst; OKC/Si: Syndromic odontogenic keratocyst; OKC/RS: Recurrent sporadic odontogenic keratocyst; OOC: Orthokeratinized odontogenic cyst; RC: Radicular cyst; DC: Dentigerous cyst; AMc: ameloblastoma, conventional; UA: ameloblastoma, unicystic; AOT: Adenomatoid odontogenic tumor.
